# Proteome analysis of human substantia nigra in Parkinson's disease

**DOI:** 10.1186/1477-5956-6-8

**Published:** 2008-02-14

**Authors:** Cornelius J Werner, Roland Heyny-von Haussen, Gerhard Mall, Sabine Wolf

**Affiliations:** 1 Department of Neurology, University Hospital RWTH Aachen, Pauwelsstr. 30, 52074 Aachen, Germany; 2 Institute of Neuroscience and Biophysics – Department of Medicine, Research Centre Juelich, 52425 Juelich, Germany; 3 Institute of Pathology, Klinikum Darmstadt, Grafenstrasse 9, 64283 Darmstadt, Germany; 4 Clemens-Schöpf-Institute for Organic Chemistry and Biochemistry, Technical University Darmstadt, Petersenstrasse 22, 64285 Darmstadt, Germany

## Abstract

**Background:**

Parkinson's disease (PD) is the most common neurodegenerative disorder involving the motor system. Although not being the only region involved in PD, affection of the substantia nigra and its projections is responsible for some of the most debilitating features of the disease. To further advance a comprehensive understanding of nigral pathology, we conducted a tissue based comparative proteome study of healthy and diseased human substantia nigra.

**Results:**

The gross number of differentially regulated proteins in PD was 221. In total, we identified 37 proteins, of which 16 were differentially expressed. Identified differential proteins comprised elements of iron metabolism (H-ferritin) and glutathione-related redox metabolism (GST M3, GST P1, GST O1), including novel redox proteins (SH3BGRL). Additionally, many glial or related proteins were found to be differentially regulated in PD (GFAP, GMFB, galectin-1, sorcin), as well as proteins belonging to metabolic pathways sparsely described in PD, such as adenosyl homocysteinase (methylation), aldehyde dehydrogenase 1 and cellular retinol-binding protein 1 (aldehyde metabolism). Further differentially regulated proteins included annexin V, beta-tubulin cofactor A, coactosin-like protein and V-type ATPase subunit 1. Proteins that were similarly expressed in healthy or diseased substantia nigra comprised housekeeping proteins such as COX5A, Rho GDI alpha, actin gamma 1, creatin-kinase B, lactate dehydrogenase B, disulfide isomerase ER-60, Rab GDI beta, methyl glyoxalase 1 (AGE metabolism) and glutamine synthetase. Interestingly, also DJ-1 and UCH-L1 were expressed similarly. Furthermore, proteins believed to serve as internal standards were found to be expressed in a constant manner, such as 14-3-3 epsilon and hCRMP-2, thus lending further validity to our results.

**Conclusion:**

Using an approach encompassing high sensitivity and high resolution, we show that alterations of SN in PD include many more proteins than previously thought. The results point towards a heterogeneous aetiopathogenesis of the disease, including alterations of GSH-related proteins as well as alterations of proteins involved in retinoid metabolism, and they indicate that proteins involved in familial PD may not be differentially regulated in idiopathic Parkinson's disease.

## Background

Parkinson's disease (PD), being the most frequent neurodegenerative motor disorder [1], is characterized by subsequent affection of brainstem structures, finally reaching cortical areas [2], by an as yet unknown pathogen or pathogenic mechanism. The severity of the clinical syndrome comprising motor disturbance (called parkinsonism) and dementia is associated largely with a reduction of striatal dopamine content, proportional to the degeneration of dopaminergic neurons in the substantia nigra. In recent years, incidence and prevalence of this debilitating disease have risen greatly [3], thus leading to an ever-rising socio-economic impact: depending on the stage of the disease, costs per year and patient varied between 4.736 € and 29.265 € in the UK [4]. Although current therapeutic regimes have lead to an increase of life expectancy up to almost normal levels in patients [5], there is no known curative therapy until now. Therefore, research on the aetiopathology of PD is of high importance.

Current research on the aetiology of PD has recognized mitochondrial and proteasome dysfunction as well as oxidative stress as major factors. Interestingly, all chemical models of PD, including 1-methyl 4-phenyl 1,2,3,6-tetrahydropyridine (MPTP) [6] and rotenone [7], lead to an increase of oxidative stress as well as mitochondrial and proteasome dysfunction. Still, they cause an acute and selective loss of dopaminergic neurons, which might not exactly mirror the pathology involved in idiopathic PD. Therefore, additional mechanisms, like inflammation, have been discussed recently [8]. While genetic forms of the disease involving mutations in genes for proteins like alpha-synuclein [9,10], parkin [11] and ubiquitin carboxyterminal hydrolase L1 (UCH-L1) [12], have shed substantial light on possible pathomechanisms such as failure of the ubiquitine-proteasome-system, these seem to account for a small minority of cases only [13]. All of these aetiologic mechanisms have been reported by numerous groups using a wide array of methods, but still it has not been possible to convert these advances in knowledge into therapeutic strategies. It therefore has been suggested recently that research on PD should move away from traditional targets and methods [14].

Previous work has shown the high potential of proteomics in analysing the molecular changes in parkinsonian brains [15,16] without the restrictions imposed by e.g., the availability of antibodies against suspected candidate proteins. Still, current data on human brain tissue are very scarce [15].

Expanding on this important work, we tried to identify expression profiles of Substantia nigra pars compacta (SNPc) in Parkinson's disease as compared to neurologically unaffected, healthy controls, using larger gels and utilizing advanced technology in staining, spot detection and picking [17]. We examined tissue samples from five patients suffering from (neuropathologically confirmed) idiopathic Parkinson's disease [18,19] without concomitant neurological or malignant disease, and compared these samples with tissue samples gained from five age- and gender-matched controls. We also controlled for the post-mortem interval (time from death to autopsy). Protein separation was done by two-dimensional polyacrylamide gel electrophoresis (2D-PAGE) followed by SyproRuby™ gel staining to visualize proteins for automated spot picking. After that we identified a number of stable and differentially expressed proteins using matrix-assisted laser desorption/ionisation time-of-flight mass spectroscopy (MALDI-ToF-MS).

In accordance with previous data, we measured a stable expression of housekeeping proteins as well as markers suitable for detecting differences in postmortem interval. Furthermore, we also detected changes in proteins regulating iron and redox metabolism as well as changes in glial proteins. These changes have been identified as hallmarks of Parkinson's disease previously. More importantly however, we identified proteins in metabolic or structural functions not yet regularly associated with idiopathic Parkinson's disease, such as enzymes critical in L-DOPA-methylation, retinoid metabolism and novel redox proteins such as SH3BGRL, thus paving the way for further research on new targets in Parkinson's disease.

## Results

### Sample homogeneity

While the ratio of male to female was exactly equal between groups (m:w = 3:2), the respective age of patients and controls had to be subjected to Fisher's exact test in order to detect any possible differences. No significant differences between groups were shown. In a similar vein, post-mortem interval data were examined. Again, no significant differences were shown. This was taken as evidence for matched groups in this regard (see Table 1 for patient data).

**Table 1:** Patient data.

***Code***	***Sex***	***Age (y)***	***post mortem interval (h)***	***Parkinson +/-***
MH2	f	94	43	-
MH3	f	89	32	-
MH4	m	66	24	-
MH6	m	64	30	-
MH7	m	74	22	-
mean ± SD.		77.4 ± 13.52	30.2 ± 8.26	
				
MH5	f	92	24	+
MH8	m	81	22	+
MH9	m	91	62	+
MH10	m	73	46	+
MH11	f	84	24	+
mean ± SD.		84.2 ± 7.79	35.6 ± 17.74	
Fisher Test p-value	0.48	0.4	0.4	
across groups	(n.s.)	(n.s.)	(n.s.)	

Patient data of both PD positive and negative samples. f = female; m = male; SD = standard deviation; Fisher Test p-value = p-value according to Fishers Exact Test (values smaller than 0.05 are considered significant), n.s. = not significant.

### Tissue preparation

**Figure 1:**
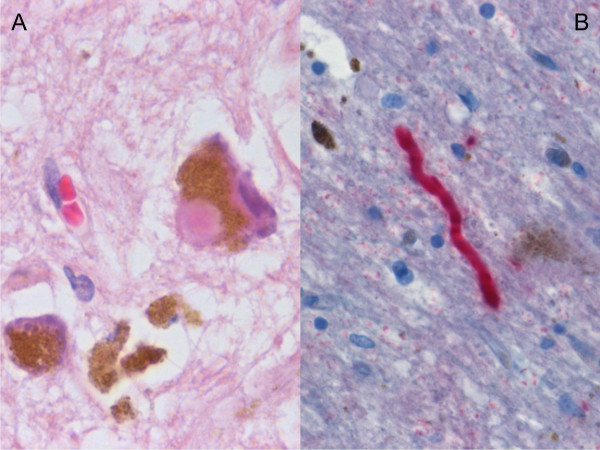
**Histological sample of Substantia nigra in Parkinson's disease**. A. SNpc neuron with a Lewy body, extracellular neuromelanin and pigment-laden macrophages. Haematoxylin/Eosin stain, 500×. B. Alpha-synuclein-positive Lewy neurit, 400×.

All specimens were classified according to current neuropathological criteria. On microscopic examination, we detected a loss of neurons in the SNpc, depigmentation of SNpc neurons, extracellular neuromelanin and clusters of pigment-laden macrophages in all cases of idiopathic Parkinson's disease. On light microscopy, only 3 of 5 cases of idiopathic PD showed Lewy bodies in the remaining neurons. Immunohistochemical examination, however, revealed Lewy bodies and Lewy neurites in all cases of idiopathic Parkinson's disease (see Figure 1).

### 2D-PAGE and data analysis

**Figure 2:**
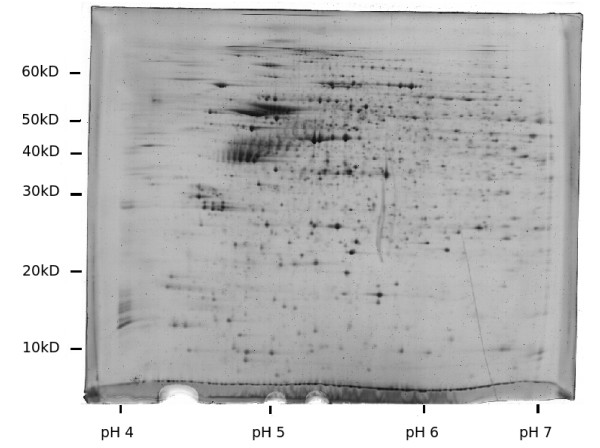
**Sample raw gel from PD group**. Representative sample. Colors are inverted and contrast is enhanced for illustration purposes. Original size 24 × 19 cm, pH 4 – 7.

**Figure 3:**
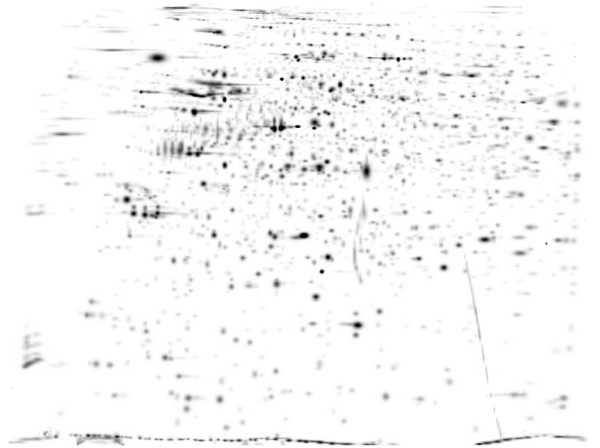
**Master gel from PD group**. Smoothed image (Gaussian kernel) of the group master gel after background removal. pH 4–7, 24 cm.

Three gels were produced of each specimen. On average, the analysis software detected 1923 (± 692) spots per gel. This is comparable to silver staining protocols and vastly superior to traditional Coomassie stains. Visual inspection yielded no significant differences in terms of spot positions or relative densities. The correlation coefficients after matching of all gels to the master gel ranged from 0.616 to 0.83 (mean 0.717), indicating a high similarity. A representative sample gel is displayed in Figure 2, while the master gel (background removed and smoothed with a Gaussian kernel) is shown in Figure 3.

**Figure 4:**
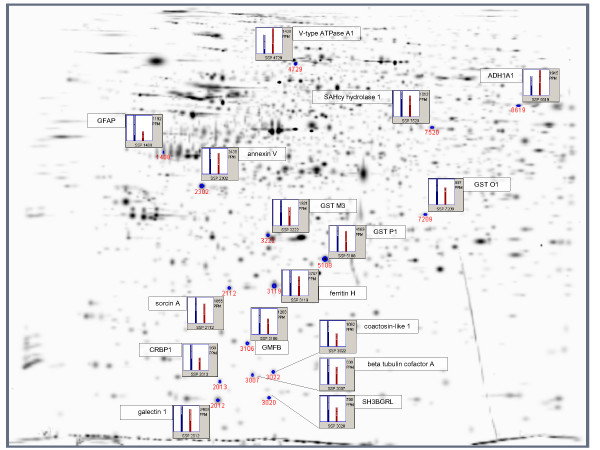
**Differentially expressed proteins**. Gaussian representation of differentially expressed spots, n = 16. ppm = parts per million, SSP = Standard Spot Number, blue bar = average density of the Parkinson group, red bar = average density of the Control group.

Statistical analysis with the Mann-Whitney Test as implemented in PDQuest 7.10 yielded 221 differentially expressed spots between groups (p ≤ 0.05). Of these, 25 spots were excised, and 16 were successfully identified with MALDI-MS. Additional file 1 lists all differentially expressed proteins. Their position on gel is shown in Figure 4.

**Figure 5:**
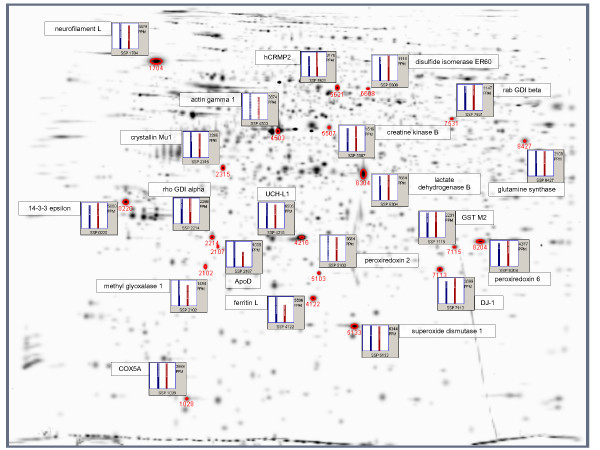
**Non-differentially expressed proteins**. Gaussian representation of non-differentially expressed spots, n = 21. ppm = parts per million, SSP = Standard Spot Number, blue bar = average density of the Parkinson group, red bar = average density of the Control group.

Apart from that, the software identified 321 spots showing a strictly conserved pattern of expression (difference less than ± 10%). Here we were able to obtain unambiguous database matches for 12 (out of 16 picked) proteins (shown in Additional file 2, marked with an asterisk [*]). Nine more proteins that were neither significantly different nor strongly conserved were also identified by MALDI-MS or electro-spray ionisation (ESI, see also Additional file 2). The respective positions can be taken from Figure 5.

Sequence coverage and accession numbers for all proteins, as well as their mean densitometric values (± standard deviation) per group and their respective Mann-Whitney p-values can be taken from Additional file 3.

Data of excised spots, for which a MALDI-MS identification failed, are not given.

Differential proteins comprised elements of iron metabolism: both ferritin H and ferritin L showed an increase of about 33%, although only ferritin H reached significance.

Furthermore, differentially expressed proteins comprised glutathione-S-transferase (GST) M3, GST P1, GST O1 and SH3-binding glutamic acid-rich like protein (SH3BGRL), all of which were expressed higher in PD than in controls. On the other hand, GST M2, peroxiredoxins 2 and 6 as well as Cu, Zn-superoxide dismutase (SOD1) showed a constant pattern of expression. Also, glial and related proteins were found to be differentially upregulated in PD, such as glial fibrillary acidic protein (GFAP), glial maturation factor beta (GMFB), galectin 1 and sorcin A. Differentially expressed structural proteins comprised V-type ATPase A1 (downregulated).

Proteins belonging to further metabolic pathways not regularly associated with PD were also found to be differentially regulated, such as S-adenosyl homocysteine (SAHcy) hydrolase 1 (L-DOPA methylation), displaying a higher densitometric profile in PD than in controls. Aldehyde dehydrogenase A1 (ADH1A1) and cellular retinol-binding protein 1 (CRBP1), both involved in aldehyde metabolism, were also differentially expressed in an increased manner as compared to controls. Additional differentially regulated proteins included annexin V, beta-tubulin cofactor A and coactosin-like protein 1.

Proteins that were similarly expressed in healthy or diseased substantia nigra comprised cytochrome c oxydase 5A (COX5A), Rho GDP dissociation inhibitor (GDI) alpha, actin gamma 1, creatine kinase B, lactate dehydrogenase B, disulfide isomerase ER-60, Rab GDI beta, apolipoprotein D (ApoD) and glutamine synthetase. Other constantly expressed proteins were 14-3-3 epsilon, crystallin Mu1, methyl glyoxalase 1 (GLO1) and human collapsin response mediator 2 (hCRMP2).

Finally, DJ-1 and UCH-L1 also belonged to the group of proteins that showed no difference in their expression profiles, both exhibiting a difference across groups of less than 10%.

### Post hoc correlation of densitometry with age and post-mortem interval

Only densitometric values of Rho GDI alpha showed a significant correlation with the post-mortem interval (r_s _= 0,7 [critical value of 0,632], p ≤ 0.05). No other proteins were significantly associated with post-mortem interval or age, respectively.

## Discussion

### Methods

Groups were matched for age and sex, as well as for the post-mortem-interval (PMI). Besides, no diabetes mellitus or malignant disease was known at the time of death in any of the specimens.

Although sample MH9 demonstrated a higher PMI than all other samples, we still believe our results to be valid, as preparative work on substantia nigra from pig brains stored for 24, 48 and 72 hours at 4°C did not show significant pattern changes (data not shown). Previous studies showed that most post-mortem changes take place during the first 24 hours [20], with only little changes after that. More importantly, however, evidence for highly comparable specimens stems from our proteome analysis itself: in our study we show similar expression profiles for classical household proteins, for which no relative difference in expression was expected. Among these, we identified creatine kinase B, lactate dehydrogenase B and glutamine synthase, the last two of which showed an densitometric difference of less than 10% across groups. Also, other non-differentially regulated proteins like COX5A, actin gamma 1, disulfide isomerase ER60, rab GDI beta, rho GDI alpha and 14-3-3 epsilon all are part of basic metabolic pathways, or they are ubiquitiously expressed structural or signalling proteins, for which a differential expression could not be expected. We also assign crystallin Mu1 to this group, as it might be implicated in cellular osmoregulation or aminoacid metabolism [21]. In addition, proteins for which a positive correlation with post-mortem interval or sample storage time (even at 4°C) had been demonstrated previously, could be shown to be expressed in a constant manner across groups. These proteins comprised hCRMP2 [22], 14-3-3 epsilon and neurofilament L [20], all of which are part of neuronal or general cell metabolism (hCRMP2 [23], 14-3-3 epsilon [24]) or structure (neurofilament L). Finally, as in our data we show a correlation with post-mortem interval for rho GDI alpha only (which is very similarly expressed across groups), we take this as evidence for a good match between groups in this regard.

### Familial Parkinson's Disease

We identified two proteins that are involved in certain forms of familial PD, namely DJ-1 and UCH-L1. Both are found to be expressed in a highly similar manner in both groups (difference of means less than 10%, respectively). This is in congruence with the results by Basso and colleagues [15], who also found a constant expression of UCH-L1 in an experiment using a similar approach to ours. Similarly, a recent genetic analysis has found no association between UCH-L1 and Parkinson's disease [25]. On the other hand, Choi and colleagues [26] described oxidative modifications and a decrease of UCH-L1 in frontal cortex of PD patients. Yet, there is the caveat that frontal and nigral pathology might differ significantly, as will be shown again below. As for DJ-1, it has been demonstrated that only one of the pathogenic mutations actually leads to a downregulation of this mitochondrial protein [27]. Therefore, mere changes in the protein level do not seem to be responsible for its pathogenic potential. Regarding our results, we do not find any evidence for a differential involvement of both DJ-1 or UCH-L1 levels in idiopathic PD.

### Neuronal Cell Death

ApoD is regarded to be a marker of neuronal cell death. Increases in protein levels have been described in Alzheimer's disease [28,29], and very recently in Parkinson's disease [30]. In our study, we find an increase of about 33% in PD, which, however, does not reach significance.

Annexin V, on the other hand, is expressed significantly higher in PD. This protein plays a role in apoptotic cell death (but also necrosis) by shielding exposed phosphatidyl serine (PS) groups [31], a function which also has been demonstrated in neuronal cell death [32]. In PD, one previous study has demonstrated a decrease in Annexin V concentrations in cerebrospinal fluid (CSF) of patients [33]. The authors argue that this was due to a consumption of Annexin V following neuronal cell death in PD. While this nicely complements our findings, it remains to be elucidated whether the amount of neuronal death in PD brains is sufficient to lead to a measurable consumption of Annexin V in CSF.

### Iron Metabolism

The involvement of iron and iron storage metabolism has been clearly established in idiopathic Parkinsons's disease, although the exact mechanisms remain unknown. Altered levels of ferritin proteins have been shown consistently in PD in a multitude of experiments [34-36]. Our results confirm these findings: both ferritin L and H are upregulated by about 33% in PD, although a statistically significant increase can be demonstrated for ferritin H only. As iron is mostly stored in nigral neuromelanin [37], it can be speculated that the upregulation of iron-storing ferritins is a compensatory mechanism against rising nigral iron levels [38] in the face of a progredient decrease in neuromelanin storage facilities.

### Glutathione and Related Enzymes/Redox Metabolism

It has been shown consistently that levels of reduced glutathione (GSH) are decreased in Parkinson's disease [39-42]. Sporadically, changes have been reported in activity (gamma-glutamyl transpeptidase, [43]) or immunoreactivity (glutathione-peroxidase, [44]) of glutahione-related enzymes. Therefore, it is a significant finding of our study that several members of a class of GSH-related detoxification enzymes are significantly increased in PD: glutathione-S-transferase Mu3 (GST M3), glutathione-S-transferase Pi1 (GST P1) and glutathione-S-transferase omega1 (GST O1) all are affected. Interestingly, GST M2, which we also identified, shows no differential regulation. The observed increase in GST M3 and GST P1 could therefore mirror the attempt of surviving neurons and glial cells to keep up redox defense mechanisms in a subenzyme-specific manner, possibly due to substrate specifity in detoxification reactions [45,46]. Particularly, polymorphisms in the GST P1 gene have been associated with PD [47,48]. Additionally, it has been demonstrated that increased levels of GST enzymes are neuroprotective in a drosophila model of PD [49], further illustrating the importance of GST enzymes in PD. We also would assign GST-omega1 to this family of proteins. Little is known about its functions, but it seems to be involved in the recycling of ascorbic acid [50], which also has antioxidant properties. Interestingly, the omega class of GSTs has been implicated in modifying the age at onset for both Alzheimer's and Parkinson's disease [51,52].

Finally, SH3-binding domain glutamic acid-rich like (SH3BGRL) could be another enzyme with antioxidant properties using GSH, because its sequence shows some analogy to *E. coli *glutaredoxin 1 [53], although its exact function again remains unknown. Structural analyses, however, seem to indicate that SH3BGRL represents a novel class of the thioredoxin fold proteins. It is thought to be involved in redox-related bioprocesses in a unique way, as it is structurally very dissimilar to the known classes of thioredoxins [54]. This finding merits further attention, as little is known about this novel enzyme class, particularly about its possible involvement in neuroprotection and Parkinson's disease. Additionally, this multitude of altered GSH-related proteins seems to warrant further research on this topic, particularly in the light of potentially GSH-preserving and neuroprotective approaches that have been described just recently [55].

In contrast to the differential expression of several members of the GST class of enzymes, we identified peroxiredoxines 2 and 6 (PRDX2, PRDX6) to be expressed in a constant manner, with PRDX6 differing less than 10% between groups. Support for our finding stems from a study performed by Power and colleagues, who showed comparable levels of immunoreactivity in SN of patients and controls [56]. Levels of PRDX6 were increased in cortical tissue of PD patients, however, again pointing towards different pathoaetiological mechanisms across the brain. Interestingly, PRDX6 has membrane-antioxidant properties [57] which depend on both GSH and GST P1 [58], the latter of which we show to be elevated in PD. It should be the aim of further studies to elucidate the relationship between these enzymes and GSH levels in Parkinson's disease.

Another enzyme with potentially important antioxidant properties is SOD1, which is implicated in certain forms of amyotrophic lateral sclerosis (ALS). Similarly to PRDX6, we find a constant level of expression. This is in congruency with previous work that demonstrated that polymorphisms in the SOD1 gene seem to play no role in both familial [59] and idiopathic PD [60]. On the other hand, a Western Blot analysis of affected cortical tissue in PD showed an increase of SOD1 [61], once more demonstrating differences in pathology between cortex and SN.

### Glial Activation

Mirroring increased glial activity in PD-affected nigral tissue [62], we show an increase of glia maturation factor beta (GMFB), which has neuroprotective properties by inducing brain derived neurotrophic factor (BDNF) [63]. GMFB also promotes neuronal and glial maturation, leading to an increase in expressed GFAP in astrocytes [64]. GFAP also is increased in PD samples in our study, thus nicely complementing the former finding. Most interestingly, we also identified a second protein with neuroprotective properties: galectin 1 (GAL1, [65]), which is overexpressed in our PD samples. Notably, galectin 1 fulfils its role by (again) inducing BDNF, but only when present in a reduced state [66], illustrating the susceptibility of this system against oxidative insults. Supplementing our findings, we show an increase of sorcin A in PD tissues. Sorcin A is involved in maintaining astrocyte homeostasis [67] and has been already been described to be elevated in PD by Jin and colleagues [16]. In summary, we find indicators of astrocytic activation, possibly exerting neuroprotective effects in an attempt to salvage remaining neurons.

### AGE Metabolism

Metabolism of advanced glycosylation endproducts (AGEs) has been debated previously in pathology of neurodegenerative diseases like Alzheimer's disease (AD) [68]. In particular, methyl glyoxalase 1 (GLO1) has been shown to be an important enzyme detoxifying AGEs [69]. In our study we show an increased expression of GLO1 in the Parkinsons's diesease group, which, however, does not reach significance. It is worth of note in this regard that the corresponding spot could not be detected in two out of five samples of the control group, thus influencing Mann-Whitney statistics. GLO1 expression levels have been shown to be associated with AD stages previously [69]. GLO1 activity, again, is limited by the availability of reduced glutathion [70]. It has been shown previously that reduced GSH levels lead to an increase in cellular damage by protein glycation [71]. AGEs themselves can be demonstrated to be associated with Lewy bodies in so-called "Incidental Lewy body disease", which is viewed as a preclinical form of PD by some authors, although this is very much a matter of debate and final proof is lacking. It could be speculated in analogy that AGE formation is part of very early processes in the course of PD [72,73]. Although we could not show a significantly differential expression of GLO1, its dependence on GSH could lead to a reduced activity. Therefore, research on AGEs and GLO1-mediated detoxification and its dependence on GSH should provide helpful insights on early PD pathology, maybe even leading to new therapeutic strategies as proposed for AD [74].

### Retinoid Metabolism

Cytosolic aldehyde dehydrogenase (ALDH1A1) has been described to be expressed specifically in dopaminergic neurons in the human substantia nigra [75]. There, it is involved in dopamine metabolism [76] and in detoxification of aldehydes such as 4-hydroxynonenal [77,78]. Therefore, it is worth of note that ALDH1A1 is significantly reduced in PD in our samples. Similar findings have been reported by several other authors using different techniques [16,79,80]. This might reflect the fact that dopaminergic neurons, of course, are reduced in number in PD, but it also constitutes an additional vulnerability against neurotoxins. In this context, it is an interesting finding that cellular retinol binding protein 1 (CRBP1) is elevated in our PD samples. CRBP1 binds retinol and retinal and transports them to specific aldehyde dehydrogenases, where e.g., retinal is metabolized to retinoat [81]. This latter agent is essentially involved in dopaminergic transmission in mice [82], therefore an increase in CRBP1 could reflect a compensatory mechanism for the aforementioned loss of ALDH1. Additionally, CRBP1 upregulation is thought to be the driving mechanism to increase retinol influx into cells [81]. Retinoids are essential in midbrain dopaminergic homeostasis, as demonstrated by Nurr1-deficient mice. Nurr1, an orphan nuclear receptor that forms heterodimers with the retinoic acid receptor RXR, has been shown to be crucial for DA neurons in the midbrain: knockout mice lacking the gene for Nurr1 fail to develop dopaminergic neurons in the mesencephalon [83]. Both findings highlight the importance of retinoid metabolism in PD, which received relatively little attention in Parkinson research until now.

### Structure and Cytoskeleton

Altered levels of structural proteins have been demonstrated in neurological and psychiatric disorders using proteomic assays before [15,16,84]. Therefore, the increase of beta tubulin cofactor A (binding to tubulin) and coactosin-like protein 1 (binding to F-actin) could be indicative of a structural reorganisation of parkinsonian substantia nigra. Additionally, we find a decrease in V-type ATPase subunit A1 (V-ATPase A1). This protein is adressed specifically to nerve terminals, where it presumably is involved in synaptic vesicle maintenance [85]. Therefore, a decrease of this synapse associated protein could nicely mirror the reduction of afferent synaptic terminals to substantia nigra, which has been described in PD using PET just recently [86], and the consequences of which have not been studied in detail so far.

### L-DOPA Methylation

The observed increase of S-adenosyl homocysteine hydrolase (SAHcy hydrolase) in the Parkinson group can be explained by an increased methylation metabolism of L-3,4-dihydroxyphenylalanine (L-DOPA), mediated by catechol-O-methyl transferases (COMTs). These enzymes use S-adenosyl-methionine (SAM) as a methyl donor, leaving S-adenosyl-homocysteine (SAH) as a residue [87]. SAH is then converted to homocysteine by the aforementioned adenosyl homocysteine hydrolase. Although seeming paradoxical at first, this increased L-DOPA methylation metabolism in PD can be explained itself by the usually applied external supplementation of L-DOPA: patients treated with L-DOPA show a decrease of SAM [88] and an increase in methylation products and homocysteine [89], while patients treated with dopamine agonists [90,91] or patients additionally treated with COMT inhibitors [92] do not exhibit such changes. We are able to demonstrate the missing link between these findings for the first time by showing an increase of an essential enzyme is this metabolic chain. Although the clinical relevance of increased homocysteine levels, a known cardiovascular risk factor, is not known currently for Parkinson's patients [93], this evidence could be taken to advocate a concurrent treatment with COMT inhibitors early in the disease, although further studies are needed on that account.

## Conclusion

Our study leads to several conclusions. First of all, we demonstrate the suitability and potential of this approach in investigating neurodegenerative diseases. We also describe a set of epiphenomena involving tissue structure and L-DOPA metabolism that can be expected given the known changes of parkinsonian substantia nigra, but which may also have clinical implications, as seen in the case of L-DOPA methylation and homocysteine levels. Beyond that, we show that a multitude of proteins could be involved in the pathogenesis of Parkinson's disease, many of which interact.

Particularly, we show that in Parkinson's disease, redox mechanisms are altered severely, but in a distinct manner. While it is obvious that GSH dependent enzymes or proteins are affected the most (with GSH independent redox enzymes being relatively spared), there seems to be a differentiation within the class of GST enzymes itself, as illustrated by the constant expression of GST-M2. This certainly warrants further investigation. It is interesting to note that the broadly recognized strain on the GSH system has impacts well beyond the known GST class of enzymes, as shown by the differential regulation of the novel protein SH3BGRL, which we demonstrate for the first time, and its possible implications for GLO1 activity. Particularly, AGE formation and detoxification and their dependance on GSH should receive further attention, as these factors could be very early contributors in the course of Parkinson's disease. We also would like to put some emphasis on examining changes in retinoid metabolism, as we are able to demonstrate the differential expression of two related proteins, both of which can be implied in dopaminergic neuronal homeostasis.

Finally, a differential expression of proteins involved in familial PD, such as DJ-1 and UCH-L1, does not seem to play a prominent role in the pathoaetiology of our samples. Therefore, research should not concentrate on familial patterns of this neurodegenerative disease alone.

Future work should expand both on examining the whole brain and focussing on subcellular fractions of idiopathic Parkinson's disease brains, as has been shown just recently [16,94]. Pursuing such an approach should shed further light on the aetiology of Parkinson's disease, hopefully paving the way to developing disease modifying or even therapeutic strategies.

## Methods

### Chemicals

Immobilized pH gradient (IPG) strips and IPG buffer were obtained from Amersham Pharmacia (Freiburg, Germany). Agarose, Bradford stain, TRIS buffer and acrylamide (Rotiphorese 30) were purchased from Roth (Karlsruhe, Germany). Alpha-cyano cinnamic acid and iodoacetamide were obtained from Sigma (Deisenhofen, Germany), CHAPS buffer was obtained from AppliChem (Darmstadt, Germany), while bromophenol blue and Sypro-Ruby were purchased from Bio-Rad (Munich, Germany). The "complete mini" proteinase inhibitor kit was provided by Roche (Mannheim, Germany) and was used as indicated by the manufacturer's instructions. All other chemicals were obtained from Merck (Darmstadt, Germany) and were used without further purification.

### Tissue specimens

Midbrain specimens were obtained with patients' informed consent in the course of regular clinical autopsies performed at the Department of Pathology, Klinikum Darmstadt. All procedures were performed according to the Helsinki Declaration. We included five specimens of clinically diagnosed and neuropathologically confirmed cases of idiopathic Parkinson's disease (3 m, 2 w, aged 84.2 ± 7.8 years, post-mortem interval [PMI] 35.6 ± 17.4 h). Current neuropathological diagnostic standards were applied [18,19]. Exclusion criteria were concurrent diseases of the CNS or malignant diseases as well as diabetes mellitus. Furthermore, we included five age- and gender-matched controls without any known neurological disease, as confirmed by autopsy (3 m, 2 w, aged 77.4 ± 13.5 years, PMI 30.2 ± 8.2 h). Age and PMI were subjected to Fisher's exact test to detect significant differences between groups.

One slide of the left part of the midbrain (2 mm thick) including parts of the left sided substantia nigra and nucleus ruber was excised for histologic examination in each case. Tissues were processed by fixation in 10% buffered formalin, followed by paraffin embedding for routine light microscopy and immunohistochemical studies.

4 μm sections were stained with haematoxylin and eosin. Immunohistochemical staining was performed by using a Ventana Benchmark automated slide stainer (Ventana, Tuscon, AZ) and an avidin-peroxidase detection kit (Ventana, Tuscon, AZ). The applied antibody was directed against alpha-synuclein (Zytomed Systems, Berlin, Germany).

The remaining tissue was frozen at -80°C until further analysis.

### Protein extraction

Substantia nigra pars compacta (SNpc) was extracted from deep-frozen brain samples using microscopic sights and ice-cooled sterile surgical instruments at a room temperature of 4°C. Tissue samples (0.125 g – 1.018 g) were homogenized by 10 strokes of an ice-cooled Dounce homogenisator with 7 μl lysis buffer (7 M urea, 2 M thiourea, 4% CHAPS, protease inhibitor "complete mini" 1×) per mg tissue. DNA desintegration was achieved by 3 cycles of sonication (30 s, with 30 s ice cooling in between). Insoluble components were removed by centrifugation at 40.000 g for 30 minutes at 4°C. Supernatants were recovered, and protein content was estimated by the method of Bradford [95].

### 2D-PAGE and data analysis

2D-PAGE was performed according to standard protocols, with some slight modifications. Three gels were produced of each specimen to ensure reproducibility. 100 μg protein were diluted to 360 ml with 7 M urea, 2 M thiourea, 4% CHAPS, 2.5% IPG buffer pH 4–7, 18 mM dithiothreitol (DTT), 1× "complete mini" protease inhibitor mix and traces of bromophenol blue. 90 μl basis buffer (7 M urea, 2 M thiourea, 4% CHAPS, 2.5% IPG buffer pH 4–7, 91 mM dithiothreitol and traces of bromophenol blue) were added. The resulting 450 μl were loaded on 24 cm IPG strips with a linear pH 4–7 by in-gel rehydration (14 h). IEF was performed in an IPG-Phor (Amersham Pharmacia) with the following protocol: 1 h at 200 V, 1 h at 500 V, 1 h at 1000 V, 53 min at a linear gradient to 8000 V and 9 h at 8000 V resulting in 77,700 Vh, with a constant temperature of 20°C. Prior to SDS-PAGE, the strips were equilibrated and alkylated 20 min each with 12.5 ml 6 M urea, 50 mM Tris-HCl pH 8.8, 30% glycerol, 4% SDS and 65 mM DTT in the first step and 260 mM iodoacetamide and traces of bromophenol blue in the second step. SDS-PAGE was performed using gels sized 25.5 × 20.5 × 0.01 cm^3^, with 12.8% acrylamid/bisacrylamid produced in a Hoefer DALT vertical casting system (Amersham Pharmacia). The second dimension was carried out in an Ettan DALT system (Amersham Pharmacia) using 5 W/gel for 50 min followed by 15 W/gel for 300 min. The run was interrupted when the bromophenol blue front reached the lower end of the gel. Gels were fixated with 10% methanol and 7% acetic acid and stained with Sypro Ruby fluorescent dye according to manufacturer's instructions. Gels were digitized using a Fujifilm FLA-5000 laser scanner (excitation wavelength 473 nm, emission wavelength ≥580 nm) with a resolution of 100 × 100 μm^2 ^and a pixel depth of 16 bit (gray values). Images were saved in TIFF format without further manipulation. Data were analyzed using the commercial PDQuest package, version 7.10 (BioRad). Background and noise were removed and gels were transformed to "Gaussian Images", fitting spots to a Gaussian distribution. Spots were quantitized by their relative volume (spot volume divided by volume of all valid spots) in ppm in order to account for any differences in total protein content. A representative master gel was chosen, to which all other gels were matched. Statistical analysis was carried out by performing the Mann-Whitney Signed Rank Test (U-Test) on each group of spots with a significance level of p ≤ 0.05 ("differential spots") within the software package PDQuest 7.10, highlighting differentially expressed spots across the groups "Parkinson" and "Controls". As PDQuest only allowed filtering for spots with a certain level of significance and did not return discrete p-values for each comparison, spot intensities also were analyzed separately with the "WinSTAT" add-in (R. Fitch Software, Bad Krozingen, Germany) for "Excel 2000" (Microsoft Deutschland GmbH, Unterschleißheim, Germany) in order to calculate precise p-values.

Additionally, spots with a highly similar expression profile were identified by highlighting spots with a expression difference of less than ± 10% ("strictly conserved spots"). Finally, a random set of similarly expressed spots were chosen across the gel in order to sample additional information on non-differentially expressed spots over the whole range of pI and MW values ("conserved spots").

### Spot excision and tryptic digestion

All differentially expressed spots with a densitometric amount of 500 ppm or more were marked for cutting. From the conserved or strictly-conserved group of spots only a random sample of about 35 spots were marked for excision. Spots were excised from a Sypro-Ruby dyed preparative gel loaded with 200 μg protein using the "Proteome Works" robotic spot cutter (BioRad). Spots were excised avoiding cross-contamination. Gel pieces were washed as follows: 1 × 20 min methanol 10%, 2 × 20 min HPLC grade water, 2 × 20 min acetonitrile 50%, 1 × 15 min acetonitrile 100% with 100 μl each per spot. The supernatant was removed, spots were dried for 5 min. Proteolysis was performed by adding 15 μl NH_4_HCO_3_ (50 nM) and 1 μl trypsin (50 ng/μl, solved in 1 mM HCl) and incubating the spots for 6 h.

### MALDI MS

Due to resource restrictions, only 25 differentially expressed (but all 34 non-differentially expressed) spots were analysed in the spectrometer. The hydrolysate was acidified with 15 μl trifluoroacetate (TFA) 0.2% and shaken for 30 min. ZipTip C18 pipette tips were equilibrated with acetonitrile 50% followed by TFA 0.1%. 10 μl peptide mixture were loaded onto the ZipTips. The peptides were eluted onto the MALDI target plate with a saturated solution of alpha-cyano-4-hydroxycinnamic acid 40% in 30% acetonitrile and 0.06% TFA 0.2%. The target plate was allowed to dry for 10 min before measurement. MALDI spectra were acquired using a "Voyager DE Pro" mass spectrometer (Perseptive Biosystems) fitted with a UV laser (wavelength 337 nm) and delayed extraction technology in reflector mode. Acceleration voltage was set to 20 kV, grid voltage percentage was set to 75% and guide wire voltage to 0.02%. Spectra were obtained by averaging 1000 laser shots, sweeping the whole plate homogeneously. Calibration was achieved by measuring a standardized peptide mixture (Sequazyme Mix 1 and Mix 2, Perseptive Biosystems) with monoisotopic *m*/*z *of 904.4681, 1296.6853, 1570.6774, 2093.0867 and 2465.1989. Additionally, autotryptic trypsin fragments with *m/z *842.50 and 2211.10 were used for calibration. In one case (protein SSP 5133), electrospray ionization (ESI) technology was employed to identify the protein.

### Database search

Peptide masses were matched against NCBI and SwissProt databases using MASCOT [96] and ProFound [97] algorithms. Peptide tolerance was set to ± 100 ppm and up to 2 missed cleavages. A protein was considered as identified when a significant hit was scored in at least one search engine and there was a large gap in score to the next best protein. In MASCOT, the sequence coverage had to equal or exceed 20% for the protein to be considered a significant hit. For the ESI data search peptide mass tolerance was set to ± 2 Da and fragment mass tolerance was set to ± 0.8 Da.

### Post-hoc analysis using Spearman's Rho

Spot volumes of all identified spots were subjected to Spearman's Rho rank order test to test for significant associations with patients' respective age and/or post mortem interval.

## Competing interests

The author(s) declare that they have no competing interests.

## Authors' contributions

CJW conceived of the study, performed tissue preparation, 2D-PAGE, mass spectrometry as well as data analysis and interpretation, and drafted the manuscript. RHvH performed autopsies and characterized substantia nigra samples neuropathologically by immunohistochemistry. GM was critically involved in data interpretation and helped to draft the manuscript. SW participated in design of the study, coordinated the study, supplied methodological expertise and helped to draft the manuscript. All authors read and approved the final manuscript.

## Supplementary Material

Additional file 1 Table of differentially expressed proteins. The following abbreviations are used: SSP = Standard Spot Number; (+) or (-) = over- and underexpression, respectively; MW = molecular weight; pI = isoelectric point; NCBI-Access.-Nr. = NCBI Accession number; Ratio = Ratio of PD positive/PD negative. Blue bar = average density of the Parkinson group, red bar = average density of the Control group. All proteins are significant with a p ≤ 0.05 (Mann-Whitney) uncorrected. Click here for file

Additional file 2 Table of conserved and strictly-conserved proteins. The following abbreviations are used: SSP = Standard Spot Number; MW = molecular weight; pI = isoelectric point; NCBI-Access.-Nr. = NCBI Accession number; Asterisk (*) = difference of means ≤ 10% ("strictly-conserved"). Blue bar = average density of the Parkinson group, red bar = average density of the Control group. Click here for file

Additional file 3 Standard Spot numbers (SSP), hit probabilities and MALDI-ToF-MS sequence coverages for all identified proteins. Additionally, mean densitometric values for each group are given as well as standard deviations and Mann-Whitney p-values. MW = molecular weight in kd; pI = isoelectric point; pT(%) = probability of a hit in percent; Score = MASCOT score; Cov(%) = sequence coverage in percent. Click here for file
