# Supplementary material for: Proteome analysis of human substantia nigra in Parkinson's disease
Source: Proteome Sci. 2008 Feb 14;6:8. doi: 10.1186/1477-5956-6-8 (PMC2265686; doi:10.1186/1477-5956-6-8)
Supplement: Additional file 1 — Table of differentially expressed proteins. The following abbreviations are used: SSP = Standard Spot Number; (+) or (-) = over- and underexpression, respectively; MW = molecular weight; pI = isoelectric point; NCBI-Access.-Nr. = NCBI Accession number; Ratio = Ratio of PD positive/PD negative. Blue bar = average density of the Parkinson group, red bar = average density of the Control group. All proteins are significant with a p ≤ 0.05 (Mann-Whitney) uncorrected. [file 1477-5956-6-8-S1.PDF]

#	SSP		Name	NCBI-Access-Nr.	MW (kd)	pI	Graph	Ratio
1	1408	+	Glial fibrillary acidic protein (GFAP)	NP_002046	49.9	5.4	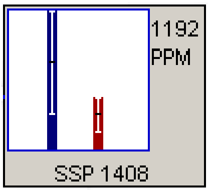	2.59
2	2012	+	galectin 1	NP_002296	15.04	5.3	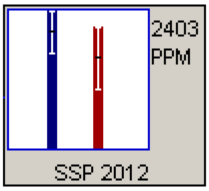	1.27
3	2013	+	cellular retinol-binding protein 1, (CRBP1)	NP_002890	16	5.0	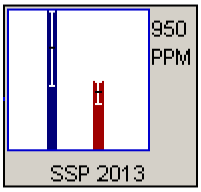	1.76
4	2112	+	sorcin A	NP_003121	21.4	5.3	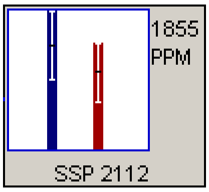	1.32
5	2302	+	annexin V	NP_001145	35.84	4.9	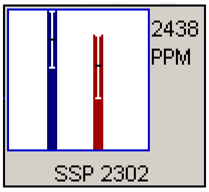	1.32
6	3007	+	beta tubulin cofactor A	NP_004598	12.89	5.3	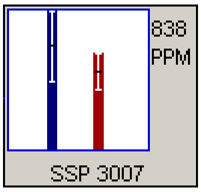	1.3
7	3020	+	SH3 domain binding glutamic acid-rich protein like (SH3BGRL)	NP_003013	12.76	5.2	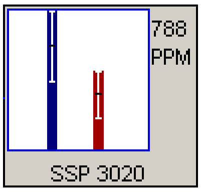	1.85

8	3022	+	coactosin-like 1	NP_066972	16.04	5.5	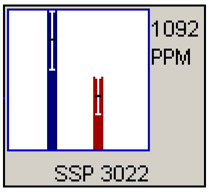	2.02
9	3106	+	glia maturation factor beta (GMFB)	NP_004115	16.87	5.2	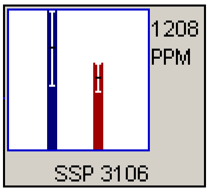	1.41
10	3119	+	ferritin heavy polypeptide 1 (ferritin H)	NP_002023	21.38	5.3	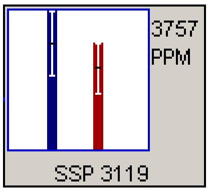	1.3
11	3222	+	glutathione-S-transferase M3	NP_000840	26.87	5.4	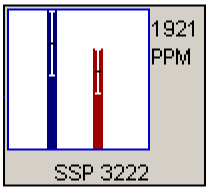	1.34
12	4729	-	V-type ATPase A1	NP_001681	68.69	5.3	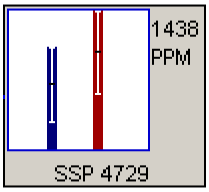	0.67
13	5108	+	glutathione-S-transferase P1	AAC13869.1	23.4	5.7	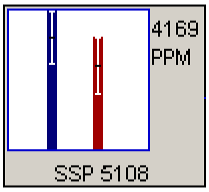	1.32
14	7209	+	glutathione-S-transferase O1	NP_004823	27.83	6.2	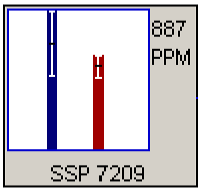	1.29

15	7520	+	S-adenosylhomo-cysteine hydrolase	NP_000678.1	48.27	6.0	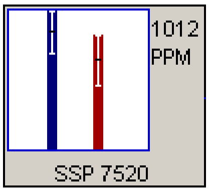	1.3
16	8619	-	Aldehyde dehydrogenase 1A1 (ADH1A1)	NP_000680	55.44	6.3	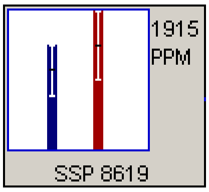	0.75
