# Supplementary material for: Proteome analysis of human substantia nigra in Parkinson's disease
Source: Proteome Sci. 2008 Feb 14;6:8. doi: 10.1186/1477-5956-6-8 (PMC2265686; doi:10.1186/1477-5956-6-8)
Supplement: Additional file 2 — Table of conserved and strictly-conserved proteins. The following abbreviations are used: SSP = Standard Spot Number; MW = molecular weight; pI = isoelectric point; NCBI-Access.-Nr. = NCBI Accession number; Asterisk (*) = difference of means ≤ 10% ("strictly-conserved"). Blue bar = average density of the Parkinson group, red bar = average density of the Control group. [file 1477-5956-6-8-S2.PDF]

#	SSP		Name	NCBI-Access-Nr.	MW (kd)	pI	Graph	Ratio
1	220	*	14-3-3 epsilon	NP_006752	29.33	4.6	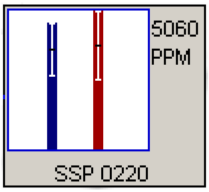	0.97
2	1028	*	cytochrome c oxidase 5A (COX5A)	NP_004246	16.93	6.3	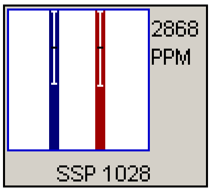	0.98
3	1704		neurofilament L	NP_006149	61.56	4.6	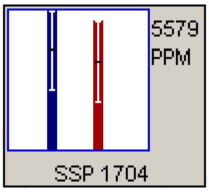	1.12
4	2102		glyoxalase 1	NP_006699	20.9	5.1	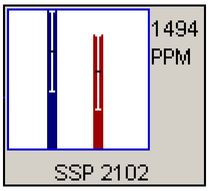	1.27
5	2107		Apolipoprotein D (ApoD)	NP_001638	21.54	5.1	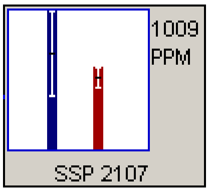	1.33
6	2214	*	rho GDP dissociation inhibitor alpha (rho GDI alpha)	NP_004300	23.25	5.0	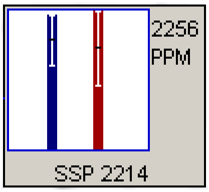	1.08
7	2315		crystallin Mu 1	NP_001879	33.93	5.1	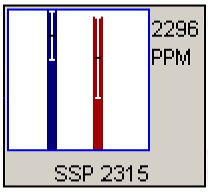	1.23

8	4122		ferritin, light polypeptide (ferritin L)	NP_000137	20.10	5.5	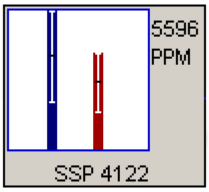	1.36
9	4216	*	ubiquitin hydrolase L1 (UCH-L1)	NP_004172	25.15	5.3	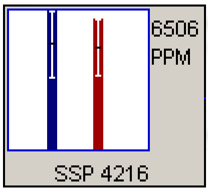	1.02
10	4503		actin gamma 1	NP_001605	41.99	5.3	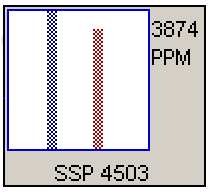	1.12
11	5103		peroxiredoxin 2 isoform A	NP_005800	22.09	5.7	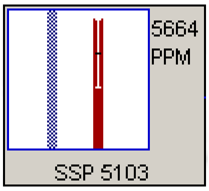	1.1
12	5133		superoxide dismutase 1 (SOD1)	NP_000445	15.88	5.7	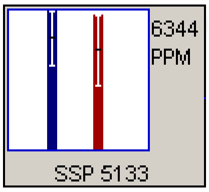	1.11
13	5507		creatine kinase B	NP_001814	42.96	5.3	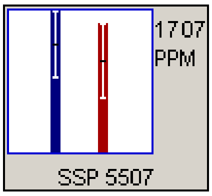	1.19
14	5621	*	human collapsin response mediator 2 (hCRMP2)	NP_001377	62.84	6.0	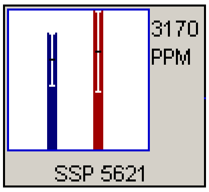	0.92

15	6304	*	lactate dehydrogenase B (LDH-B)	NP_002291	36.84	5.7	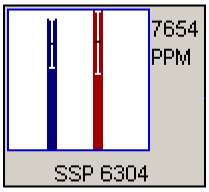	1
16	6608	*	disulfide isomerase ER60	NP_005304	57.16	2.4	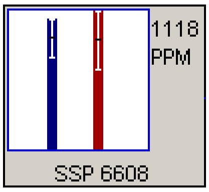	1.02
17	7113	*	DJ-1	NP_009193	20.04	6.3	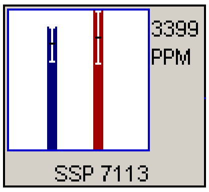	0.94
18	7115	*	glutathione-S-transferase M2	NP_000839.1	25.9	6.0	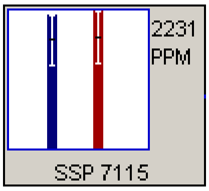	0.98
19	7531	*	rab GDP dissociation inhibitor beta (rab GDI beta)	NP_001485	51.10	6.1	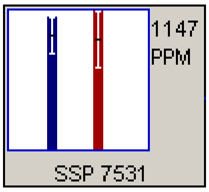	1.05
20	8204	*	peroxiredoxin 6	NP_004896	25.10	6.0	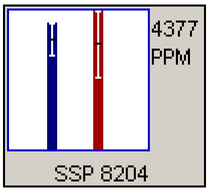	1.05
21	8427	*	glutamine synthetase	NP_002056	42.91	6.4	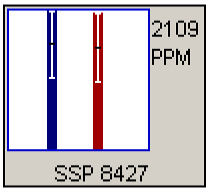	1.03
