# Supplementary material for: Proteome analysis of human substantia nigra in Parkinson's disease
Source: Proteome Sci. 2008 Feb 14;6:8. doi: 10.1186/1477-5956-6-8 (PMC2265686; doi:10.1186/1477-5956-6-8)
Supplement: Additional file 3 — Standard Spot numbers (SSP), hit probabilities and MALDI-ToF-MS sequence coverages for all identified proteins. Additionally, mean densitometric values for each group are given as well as standard deviations and Mann-Whitney p-values. MW = molecular weight in kd; pI = isoelectric point; pT(%) = probability of a hit in percent; Score = MASCOT score; Cov(%) = sequence coverage in percent. [file 1477-5956-6-8-S3.PDF]

SSP	NCBI- Access-Nr.	*Parkinson (ppm)*		*Controls (ppm)*		p (Mann-Whitney)	MW (kd)	pI	ProFound		MASCOT	
		Mean	Standard Deviation	Mean	Standard Deviation				p(T) (%)	Z-Factor	Score	Cov. (%)
220	NP_006752	4926,36	1290,92	5060,43	1678,00	0,8519	29,33	4,6	100	1,93	53	35
1028	NP_004246	2730,84	1021,19	2784,81	1004,96	0,6334	16,93	6,3	100	1,76	63	33
1408	NP_002046	1191,58	708,09	460,47	241,36	0,0025	49,9	5,4	100	2,39	331	39
1704	NP_006149	5578,87	2275,30	4965,91	2326,38	0,9504	61,56	4,6	100	2,43	129	23
2012	NP_002296	2403,08	436,58	1886,17	656,79	0,0294	15,04	5,3	100	2,35	103	47
2013	NP_002890	949,97	351,64	541,23	107,60	0,0002	16	5	100	2,43	100	55
2102	NP_006699	1494,07	627,65	1176,84	575,17	0,4561	20,9	5,1	100	2,43	93	41
2107	NP_001638	1009,34	446,06	758,89	111,39	0,0896	21,54	5,1	100	2,43	X	X
2112	NP_003121	1855,48	628,46	1401,34	533,55	0,0238	21,94	5,3	100	2,37	86	35
2214	NP_004300	2443,55	580,13	2255,63	844,64	0,4679	23,25	5	100	2,37	102	33
2302	NP_001145	2438,01	646,44	1849,33	697,38	0,0171	35,84	4,9	100	2,37	151	37
2315	NP_001879	2296,49	488,03	1861,61	813,77	0,0930	33,93	5,1	100	2,37	104	40
3007	NP_004598	838,11	294,87	642,94	149,32	0,0281	12,89	5,3	100	2,43	90	36
3020	NP_003013	788,00	271,35	425,25	178,20	0,0004	12,76	5,2	100	1,18*	80	35
3022	NP_066972	1092,08	297,03	540,53	191,53	0,0001	16,04	5,5	100	2,09	115	45
3106	NP_004115	1207,79	453,85	858,16	175,86	0,0362	16,87	5,2	100	2,43	X	X
3119	NP_002023	3756,71	1151,59	2895,49	920,07	0,0362	21,38	5,3	100	2,43	105	39
3222	NP_000840	1921,47	601,42	1429,49	421,06	0,0265	26,87	5,4	100	2,43	166	59
4122	NP_000137	5595,53	2777,31	4101,53	1782,01	0,1776	20,1	5,5	100	2,43	71	32
4216	NP_004172	6506,34	2100,39	6379,97	1790,84	0,9835	25,15	5,3	100	2,43	90	46
4503	NP_001605	3873,61	2855,67	3455,65	2423,04	0,8614	41,99	5,3	100	2,0	105	32

SSP	NCBI- Access-Nr.	*Parkinson (ppm)* Mean	Standard Deviation	*Controls (ppm)* Mean	Standard Deviation	p (Mann-Whitney)	MW (kd)	pI	ProFound p(T) (%)	Z-Factor	MASCOT Score	Cov. (%)
4729	NP_001681	959,28	559,06	1438,46	616,05	0,0362	68,69	5,3	100	2,21	149	29
5103	NP_005800	5664,03	1681,05	5126,70	1823,42	0,3297	22,09	5,7	100	2,43	97	32
5108	AAC13869.1	4169,31	1009,86	3166,71	1092,24	0,0191	23,4	5,7	100	1,74	100	61
5133	NP_000445	6344,05	1585,94	5729,54	2051,04	0,7244	15,88	5,7	identified	by ESI	93	1 Peptide
5507	NP_001814	1706,51	568,59	1433,21	644,13	0,4429	42,96	5,3	100	2,34	74	32
5621	NP_001377	2908,62	880,89	3170,49	1320,79	0,6334	62,84	6	100	2,28	130	27
6304	NP_002291	7653,73	1800,55	7654,32	2307,43	0,8519	36,84	5,7	100	2,39	34	160
6608	NP_005304	1135,30	205,45	1117,81	302,83	0,8195	57,16	2,38	100	2,38	232	36
7113	NP_009193	3199,63	539,59	3398,55	794,71	0,4679	20,04	6,3	100	2,37	74	31
7115	NP_000839	2193,89	504,59	2231,06	525,48	0,9174	25,89	6	100	2,39	113	44
7209	NP_004823	886,69	267,71	686,23	99,40	0,0356	27,83	6,2	100	2,25	72	30
7520	NP_000678.1	1012,11	183,98	777,07	220,38	0,0075	48,27	6	100	2,36	186	35
7531	NP_001485	1202,52	201,71	1147,06	300,93	0,5755	51,1	6,1	100	2,29	88	29
8204	NP_004896	4578,05	697,41	4377,07	1377,74	0,5755	25,1	6	100	2,43	165	55
8427	NP_002056	2109,10	688,06	2054,75	709,27	0,8195	42,91	6,4	100	2,36	106	21
8619	NP_000680	1435,88	476,88	1915,19	633,25	0,0327	55,44	6,3	100	2,19	125	30
